# Identification and characterization of miRNAs in two closely related C_4_ and C_3_ species of Cleome by high-throughput sequencing

**DOI:** 10.1038/srep46552

**Published:** 2017-04-19

**Authors:** Shuangcheng Gao, Wei Zhao, Xiang Li, Qingbo You, Xinjie Shen, Wei Guo, Shihua Wang, Guoan Shi, Zheng Liu, Yongqing Jiao

**Affiliations:** 1 College of Agriculture, Henan University of Science and Technology, Luoyang, Henan Province, 471003, P. R. China; 2 Key Laboratory of Biology and Genetic Improvement of Oil Crops, Ministry of Agriculture, Oil Crops Research Institute of the Chinese Academy of Agricultural Sciences, Wuhan, 430062, P. R. China; 3 College of Life Sciences, Hebei University, Baoding, Hebei Province, 071002, P. R. China

## Abstract

*Cleome gynandra* and *Cleome hassleriana*, which are C_4_ and C_3_ plants, respectively, are two species of Cleome. The close genetic relationship between *C. gynandra* and *C. hassleriana* provides advantages for discovering the differences in leaf development and physiological processes between C_3_ and C_4_ plants. MicroRNAs (miRNAs) are a class of important regulators of various biological processes. In this study, we investigate the differences in the characteristics of miRNAs between *C. gynandra* and *C. hassleriana* using high-throughput sequencing technology. In total, 94 and 102 known miRNAs were identified in *C. gynandra* and *C. hassleriana*, respectively, of which 3 were specific for *C. gynandra* and 10 were specific for *C. hassleriana*. Ninety-one common miRNAs were identified in both species. In addition, 4 novel miRNAs were detected, including three in *C. gynandra* and three in *C. hassleriana*. Of these miRNAs, 67 were significantly differentially expressed between these two species and were involved in extensive biological processes, such as glycol-metabolism and photosynthesis. Our study not only provided resources for *C. gynandra* and *C. hassleriana* research but also provided useful clues for the understanding of the roles of miRNAs in the alterations of biological processes in leaf tissues during the evolution of the C_4_ pathway.

C_4_ photosynthesis, which is a complex biological trait that enables plants to accumulate biomass at a much faster rate, has evolved independently multiple times within the angiosperms^1^,^2^. Considering that C_4_ crops have greater water and nitrogen use efficiency and photosynthesis efficiency than C_3_ crops, it has been suggested that integrating the characteristics of the C_4_ pathway into C_3_ crops could be used to increase yield^3^,^4^. However, other than the basic biochemistry, we still have a very limited understanding of C_4_ photosynthesis. Particularly, the genetic basis associated with the alterations in the cell biology and development of the C_4_ leaf is poorly understood^5^. Thus, the application of integrating the C_4_ pathway into C_3_ crops to increase yield remains restricted.

The Cleome species belongs to the family Cleomaceae, which was formerly Capparaceae^6^. Capparaceae was referred to as a sister to Brassicaceae and possessed a number of C_4_ species^7^. The genus Cleome contains a phylogenetic progression from C_3_ to C_4_ photosynthesis, which provides the potential to understanding the genetic changes between the C_4_ and C_3_ photosynthetic pathways during the evolutionary process^7^. *Cleome gynandra* (also known as *Gynandropsis gynandra*) and *Cleome hassleriana* (also known as *Tarenaya hassleriana*) are two typical species of Cleome. *C. gynandra* is a C_4_ photosynthesis species, while *C. hassleriana* is a C_3_ species. Phylogenetically, both *C. gynandra* and *C. hassleriana* are near relatives of *Arabidopsis thaliana*, which is a well-known C_3_ model plant^5^,^7^. *C. gynandra, C. hassleriana* and *A. thaliana* have high sequence similarities^8^. Approximately 70% of the RNA-seq reads of *C. gynandra* and *C. hassleriana* matched approximately 55.3% of the genes in the *A. thaliana* reference^8^. Therefore, the vast molecular data and well-annotated genome resources available for *A. thaliana* can be applied to *C. gynandra* and *C. hassleriana*. Moreover, these two species have small statures and short life cycles, are self-fertile and produce a large amount of seed^6^. These advantages make *C. gynandra* and *C. hassleriana* potential C_4_ and C_3_ models for identifying the differences in leaf development and physiological processes between C_3_ and C_4_ plants.

MicroRNAs are a class of 18–24 nucleotide (nt) small non-coding endogenous RNAs that are capable of regulating gene expression at the post-transcriptional level^9^,^10^,^11^,^12^. Research regarding *A. thaliana, Oryza sativa* and maize showed that plant miRNAs played important roles in regulating plant development^13^,^14^. For example, miR165 directs the cleavage of *REV* to regulate leaf morphogenesis^15^; miR319 regulates leaf senescence by controlling the *TCP* transcription factors^10^,^16^; and miR166 targets *Leafbladeless1 (LBL1*) to influence the specification of the adaxial/upper leaf surface^17^. The identification and characterization of miRNAs would provide helpful information for understanding the biological processes in plants.

Due to the merits of *Cleome gynandra* and *Cleome hassleriana* in studying the genetic basis of the differences in the cell biology and development between C_4_ and C_3_ plants, we used high-throughput sequencing technology to identify and characterize the miRNAs in the leaf tissues of these two species to gain some insights into the molecular changes that occur during the evolution of C_3_ plants to C_4_ plants. Thus, a number of known and novel miRNAs were detected in the two species, and the expression patterns of these miRNAs were profiled. By comparing our data with published data from *Arabidopsis*, the potential functions of these miRNAs were also investigated. Our study not only provided resources for studying *C. gynandra* and *C. hassleriana* but also provided useful clues for the understanding of the roles of the miRNAs in the differences in the biological processes in leaf tissues during the evolution of the C_4_ pathway.

## Results

### Overview of small RNA sequencing data

To identify the miRNAs in *C. gynandra* and *C. hassleriana*, two small RNA libraries were constructed for the fully expanded leaves (approximately 5 cm in length) of these two species and then sequenced by an Illumina HiSeq 2500 analyzer. In total, 6,698,595 and 7,177,444 raw reads were obtained (Table 1). The raw reads were further cleaned and trimmed by removing the low-quality reads and sequences that were either smaller than 18 nt or longer than 30 nt. Then, the clean reads were aligned with the Silva, GtRNAdb, Rfam and Repbase databases to filter the ribosomal RNA (rRNA), transfer RNA (tRNA), small nucleolar RNA (snoRNA) and the repeats, which in total, accounted for approximately 45.32% and 46.68% of the clean reads from the two libraries. Finally, a total of 1,761,554 and 1,687,208 clean unannotated reads were obtained for the two libraries (Table 1).

**Figure 1:**
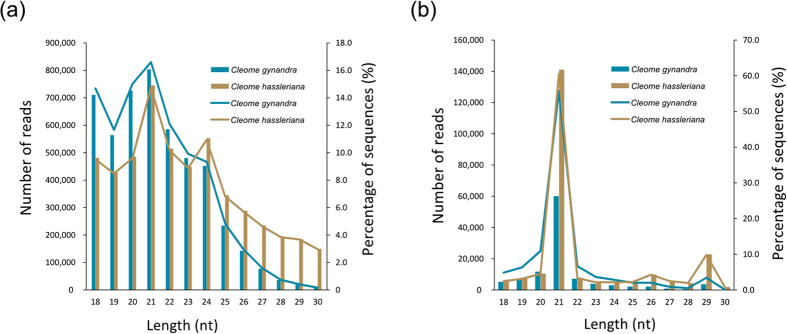
Length distribution and abundance of the small RNA sequences in *Cleome gynandra* and *Cleome hassleriana*. Percentage (line) and reads number (bar) of redundant (**a**) and mapped unique (**b**) sequences of 18–30 nt length for each sequenced library.

We investigated the length distribution of the small RNA reads (Fig. 1a and b). The most abundant small RNA in *C. gynandra* and *C. hassleriana* was 21 nt in length, accounting for 55.78% and 60.58% of total sRNA reads, respectively. The second most abundant small RNA in *C. gynandra* was 20 nt, representing 10.91% of the total sRNA reads. In comparison, the second most abundant small RNA in *C. hassleriana* was 29 nt, representing 9.81% of total sRNA reads (Supplementary Table 1).

### Identification of known miRNAs in *C. gynandra* and *C. hassleriana*

*C. gynandra* and *C. hassleriana* are members of *Cleome*, which is the most closely related genus to *Arabidopsis thaliana*^5^,^7^. Previous studies showed that *C. gynandra, C. hassleriana* and *Arabidopsis thaliana* had high sequence similarities^8^. We could use the well-annotated genome of *Arabidopsis thaliana* to identify the miRNAs and further investigate their expression in *C. gynandra* and *C. hassleriana*^18^.

The high-quality unannotated sRNA reads, which ranged from 18 to 30 nt, were aligned against the *Arabidopsis thaliana* genome. The mapped reads were further aligned with miRBase21.0 to identify the known miRNAs. In total, 94 and 102 known miRNAs were identified in *C. gynandra* and *C. hassleriana*, respectively, which were members of 32 plant conserved families (Table 2). Ninety-one known miRNAs were commonly identified in both *C. gynandra* and *C. hassleriana* (Table 2). Among these miRNA families, MIR156 contained the most abundant members, including nine members in *C. hassleriana* and eight members in *C. gynandra*. The second most abundant was the MIR166 family, which contained eight members in *C. hassleriana* and seven members in *C. gynandra*. Certain miRNA families were only detected in one species. For example, MIR397 and MIR828 were specific to *C. hassleriana*, while MIR161 and MIR8175 were specific to *C. gynandra* (Supplementary Fig. 1).

Based on the number of sequencing reads, the miRNA families displayed a significantly varied abundance (Table 2). The miRNA families MIR159, MIR164, MIR166, MIR167 and MIR168 showed a higher expression level in both *C. gynandra* and *C. hassleriana*. MIR156, MIR157, MIR390, MIR393 and MIR396 also showed an average expression level of more than 1,000 TPM in both species. In contrast, certain miRNA families, such as MIR161, MIR170, MIR171, MIR395, MIR397 and MIR399, were observed to have a low expression level (Table 2).

### Identification of novel miRNAs in *C. gynandra* and *C. hassleriana*

To investigate the novel miRNAs in *C. gynandra* and *C. hassleriana*, the unannotated sRNA sequences were used to predict novel miRNA by exploring the hairpin structures based on comparisons with the genome sequence of *Arabidopsis thaliana*, the Dicer cleavage site and the minimum free energy using miREvo and mirdeep2 software. Finally, only four novel miRNA candidates were identified and temporarily named in the format of Cleome-novel-miR-number (Table 3). Cleome_novel_miR1 and Cleome_novel_miR2 were detected in both *C. gynandra* and *C. hassleriana*. Cleome_novel_miR3 was specific to *C. gynandra*, and Cleome_novel_miR4 was specific to *C. hassleriana*. These novel miRNAs showed a relatively low expression level in both species (Table 3).

### Differentially expressed miRNAs between *C. gynandra* and *C. hassleriana*

**Figure 2:**
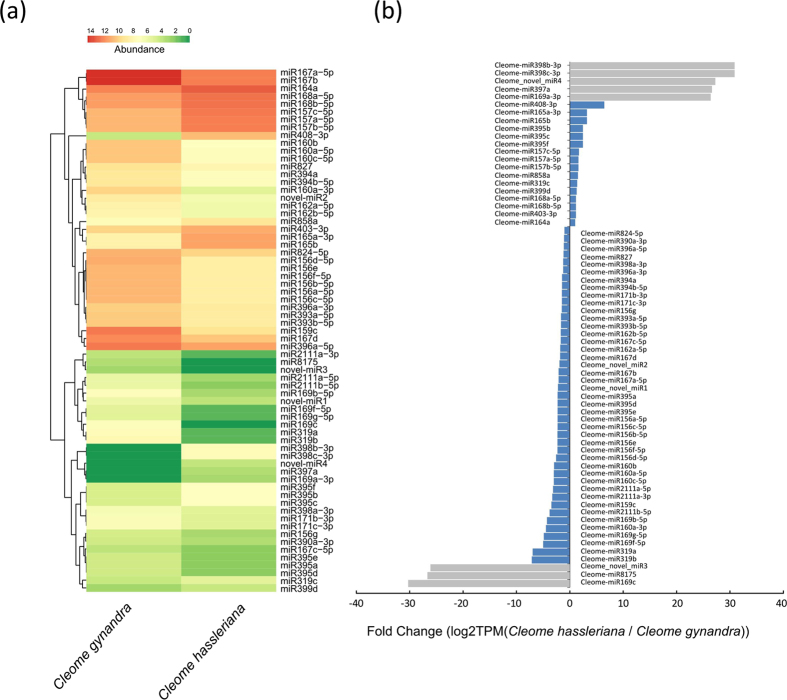
Expression patterns of miRNAs between *Cleome gynandra* and *Cleome hassleriana*. (**a**) Hierarchical cluster analysis of differentially expressed miRNAs between *Cleome gynandra* and *Cleome hassleriana*. The abundance used in the heatmap refers to the high (in red) and low (in green) expression levels of the miRNAs (TPM value of each miRNA). (**b**) Expression patterns of the differentially expressed miRNAs between *Cleome gynandra* and *Cleome hassleriana*. The color scales indicate the species-specific expressed miRNAs (grey bar) and non-species-specific expressed miRNAs (blue bar).

In total, sixty-seven differentially expressed miRNAs were identified between *C. gynandra* and *C. hassleriana*, 21 of which were down-regulated and 46 were up-regulated in *C. gynandra* compared with those in *C. hassleriana* (Fig. 2a and b).

Among these miRNAs, some showed drastically changed expression patterns. For example, the expression levels of two members of the miR165 family, Cleome-miR165a-3p and Cleome-miR165b, were both reduced nearly ten-fold in *C. gynandra* compared with those in *C. hassleriana* (Supplementary Table 2); Cleome-miR159c had a greater than ten-fold higher expression level in *C. gynandra* than that in *C. hassleriana* (Supplementary Table 2). Certain miRNAs were expressed in a species-specific manner. For example, Cleome_novel_miR3, Cleome-miR8175 and Cleome-miR169c were only detected in *C. gynandra*, while Cleome-miR169a-3p, Cleome-miR397a, Cleome_novel_miR4, Cleome-miR398b-3p and Cleome-miR398c-3p were only expressed in *C. hassleriana* (Fig. 2b and Supplementary Table 2). Some miRNAs that belong to the same miRNA family exhibited different expression patterns. For example, three members of the MIR395 family, Cleome-miR395a, Cleome-miR395d, and Cleome-miR395e, were up-regulated in *C. gynandra*, while three other members of this family, Cleome-miR395b, Cleome-miR395c and Cleome-miR395f, were down-regulated (Supplementary Table 2).

We identified 194 putative target genes for the differentially expressed miRNAs that were identified in our study (Supplementary Table 3). Külahoglu *et al*. (2014) had investigated the dynamic changes in the gene expression levels during leaf development between *C. gynandra* and *C. hassleriana* by high-throughput sequencing^19^. We analyzed these publicly available RNA-seq datasets to explore the expression levels of our putative target genes. In total, 51 of the 194 genes were found to be differentially expressed between *C. gynandra* and *C. hassleriana*, 38 of which had opposite miRNAs expression patterns between these two species (Supplementary Table 4). These 38 target genes were found to be the targets of 8 conserved and 1 putative novel miRNA families, including Cleome-miR156a-5p, Cleome-miR160a-5p, Cleome-miR164a, Cleome-miR171b-3p, Cleome-miR319c, Cleome-miR394a, Cleome-miR395a, Cleome-miR396a-5p and Cleome_novel_miR1 (Supplementary Table 4).

### Function analysis of the identified miRNAs

To investigate the roles of the miRNAs in *C. gynandra* and *C. hassleriana*, the putative target homolog genes of these miRNAs in *Arabidopsis thaliana* were predicted and analyzed using miRNA family assignment. Two hundred twenty-eight putative target genes were predicted for 68 miRNAs in *C. gynandra*. In addition, two hundred twenty-three target genes were predicted for 72 miRNAs in *C. hassleriana* (Table 4). Of the known miRNAs, the MIR156 family could target the most abundant genes, which has 15 putative target genes. The second most abundant family was the MIR161 family, which could match 13 putative target genes. Of the novel miRNAs, only Cleome_novel_miR1 and Cleome_novel_miR3 were predicted to have target genes. Notably, the novel miRNA, Cleome_novel_miR1, could match 129 putative target genes (Supplementary Table 3). No target genes were found for the remaining 29 miRNAs in *C. gynandra* and 33 miRNAs in *C. hassleriana*.

**Figure 3:**
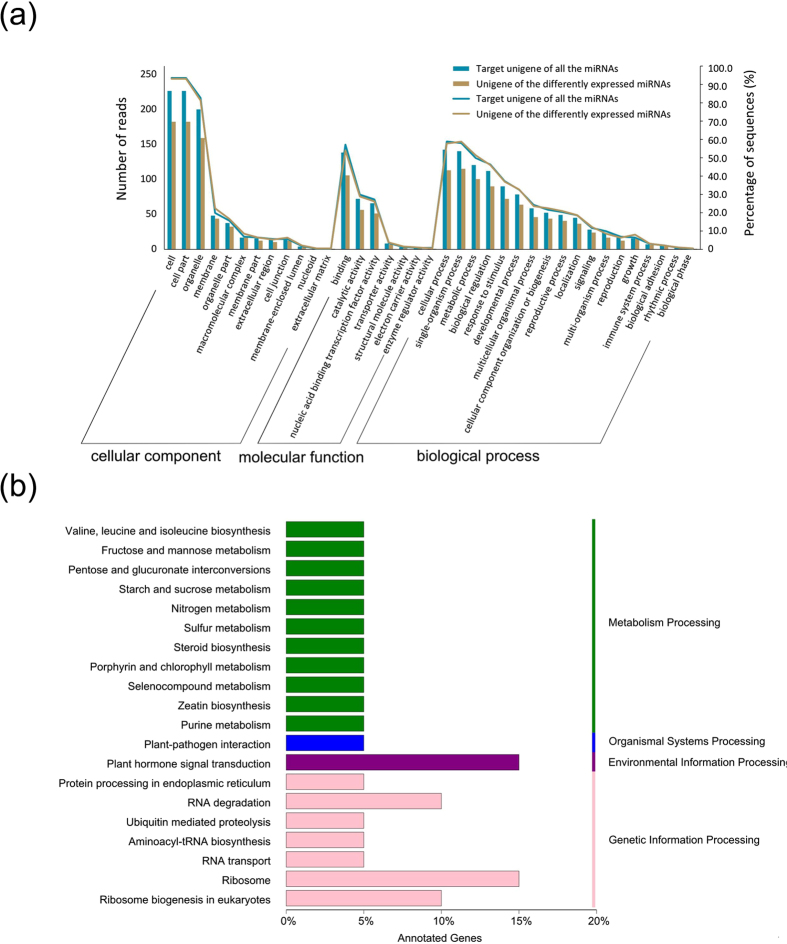
GO categories and KEGG pathway analysis for the putative target genes of the differentially expressed miRNAs in *Cleome gynandra* and *Cleome hassleriana*. (**a**) GO categories for the putative target genes of the differentially expressed miRNAs involved in biological processes, cellular components and molecular functions. (**b**) KEGG pathway analysis for the putative target genes of the differentially expressed miRNAs involved in processes.

GO categories and KEGG pathway analyses were performed for the differentially expressed miRNAs between *C. gynandra* and *C. hassleriana* based on the annotations of their putative target homolog genes in *Arabidopsis thaliana*. For the biological processes, the enriched GO terms included cellular process, single-organism process, metabolic process, biological regulation, response to stimulus, etc. For the molecular function, these target genes were mainly enriched in binding, catalytic activity, and nucleic acid binding transcription factor activity. For the cell component category, the target genes were mainly involved in cell, cell part and organelle part (Fig. 3a). The KEGG pathway analysis showed that these target genes were enriched in twenty pathways, including pathways for plant hormone signal transduction, ribosome, starch and sucrose metabolism, nitrogen metabolism, porphyrin and chlorophyll metabolism, and sulfur metabolism (Fig. 3b).

### Quantitative real-time PCR of miRNAs

**Figure 4:**
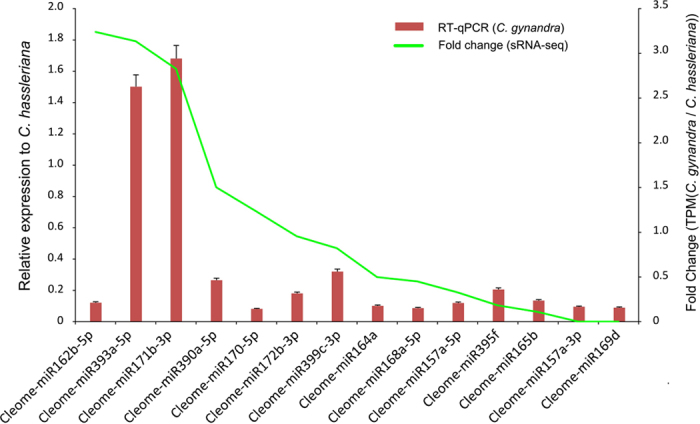
Quantitative analysis of the fourteen miRNAs levels by poly-A tail extension qRT-PCR in the leaf of *Cleome gynandra* and *Cleome hassleriana*. U6 was used as the internal control. The line represents the fold change of the expression level by sRNA-seq.

To confirm the expression patterns of the differentially expressed miRNAs that were obtained from the sequencing analysis, we confirmed the expression levels of fourteen miRNAs using quantitative real-time PCR (qRT-PCR) and compared the differences in their expression levels between *C. gynandra* and *C. hassleriana* (Fig. 4). The results showed that 13 of the14 miRNAs had expression patterns that were similar to those of the sequencing data, which indicated that our sequencing data were reliable.

## Discussion

In this study, we used two closely related C_4_ and C_3_ species, *C. gynandra* and *C. hassleriana*, to explore the genetic difference between C_4_ and C_3_ plants using high-throughput sequencing technology. Two small RNA libraries were constructed and sequenced. The obtained small RNA sequences were between 18–29 nt in length. Generally, the 21 nt small RNAs represent typical, mature miRNAs in plants. The most abundant small RNAs in *C. gynandra* and *C. hassleriana* were 21 nt in length, accounting for 55.78% and 60.58% of the total small RNAs, respectively. This result was consistent with that in the maize leaf^20^. The second most abundant sRNA in *C. gynandra* was 20 nt in length, while the second most abundant sRNA in *C. hassleriana* was 29 nt in length (Table S2). In some plants, such as soybeans, peanuts, sweet oranges and rice, the lengths of the most and second most abundant small RNAs were 24 nt and 21 nt, respectively^21^,^22^,^23^,^24^,^25^. The different length distributions of the small RNAs indicate the different characteristics of the small RNAs among various plant species.

MiRNAs are important post-transcriptional regulators of gene expression and play important roles in biological processes, including plant development^10^,^15^,^17^,^26^,^27^,^28^. Since the first plant miRNAs were reported in *Arabidopsis thaliana* in 2002, various miRNAs have been identified^29^. For example, Zhao *et al*. (2010) reported 75 conserved miRNAs and 14 novel miRNAs in peanuts^21^; Kang *et al*. (2012) detected 125 and 127 known miRNAs in maize seeds and leaves, respectively^20^; and Wang *et al*. (2015) discovered 95 known miRNAs and 23 novel miRNA candidates in cotton seeds^30^. Here, in our study, 94 known and 3 novel miRNAs were identified in *C. gynandra*, and 102 known and 3 novel miRNAs were identified in *C. hassleriana*. Ninety-one known and 2 novel miRNAs were commonly detected in both species. Our study is the first to report the identification of miRNAs in *C. gynandra* and *C. hassleriana*, which are valuable additions to the plant miRNA kingdom.

Some miRNAs in our study showed species-specific expression patterns. For example, Cleome-miR161.2 was present only in *C. gynandra*, and Cleome-miR397a was present only in *C. hassleriana* (Table 2). MiR161 has two forms, miR161.1 and miR161.2, which target *PPR-P* and regulate cytoplasmic male sterility^31^,^32^,^33^. OsmiR397 targets the laccase-like gene *OsLAC* and affects grain size and panicle branching^34^. The roles of these species-specific miRNAs in the development and function of leaf tissues in *C. gynandra* and *C. hassleriana* need to be investigated in the future.

In this study, most of the miRNAs, approximately 61.5%, were differentially expressed between *C. gynandra* and *C. hassleriana*. Of these miRNAs, some were reported to participate in the cell biology and development process of the leaf. For example, the MIR165 family directly cleaves the adaxial identity genes *REV (AT5G60690*) and *PHB (AT2G34710*) and was involved in the process of leaf morphogenesis^15^,^35^. In our study, the expression levels of two members of the MIR165 family, Cleome-miR165a-3p and Cleome-miR165b, were both reduced nearly ten-fold in *C. gynandra* (Supplementary Table 2). This expression pattern might be associated with the specified morphogenesis of the C_4_ leaf in *C. gynandra*. miR164 mediates the cleavage of *NAC1 (AT1G56010*) and *NAC2 (AT5G39610*), which were involved in the processes of lateral root emergence and aging-induced cell death and leaf senescence, respectively^36^,^37^. The over-expression of ath-miR164a repressed the EIN3-induced early senescence phenotypes in *Arabidopsis thaliana*^38^. Whether miR164 still functions as an aging regulator or has a new function in the C_4_ pathway needs to be further investigated. We also found that some miRNAs that belong to the same miRNA family have different expression patterns between *C. gynandra* and *C. hassleriana*. For example, the Cleome-miR395a/d/e was significantly up-regulated, while Cleome-miR395 b/c/f was significantly down-regulated in *C. gynandra* compared with those in *C. hassleriana* (Supplementary Table 2). The target genes of miR395 include *GUN5 (AT5G13630*), *APS1 (AT3G22890*) and *AST68 (AT5G10180*), which were reported to be involved in chlorophyll synthesis, the abscisic acid (ABA) pathway, and the sulfate metabolism pathway^38^,^39^,^40^. The diverse expression patterns of miRNAs suggested an orchestrated temporal and spatial regulation of the gene expression levels in various biological processes between these two species at the post-transcriptional level. One hundred ninety-four putative target genes were predicted for the identified miRNAs in our study (Supplementary Table 3). Among them, 38 were confirmed to have expression patterns that were opposite to those of the miRNAs in the publicly available data^19^, which indicated that these 38 genes might be regulated by miRNAs (Supplementary Table 4). We investigated the expression patterns of the putative target genes mentioned above and found that the target of miR164, *NAC1 (AT1G56010*), and the two targets of miR395, *APS1 (AT3G22890*) and *AST68 (AT5G10180*), were among the 38 genes that had expression patterns that were opposite to those of the miRNAs in the published data (Supplementary Table 4). However, the two targets of miR165, *REV (AT5G60690*) and *PHB (AT2G34710*), one target of miR164, *NAC2 (AT5G39610*), and one target of miR395, *GUN5 (AT5G13630*), had no such expression patterns. The miRNA-mediated gene regulation is complicated, which means that it might not cause the down-regulation of the target genes, e.g., translational inhibition^41^. In addition, different leaf samples and growth conditions were applied in the previous and our experiments. These differences might be the reasons why large numbers of target genes were not found to be differentially expressed or have opposite miRNAs expression patterns. To obtain more reliable results, small RNAome, transcriptome and degradome sequencings must be simultaneously conducted using the same leaf samples in the future.

The functions of differentially expressed miRNAs were investigated according to the annotation information for *Arabidopsis* (Table 4). However, there were still 29 miRNAs in *C. gynandra* that were not predicted to have target genes. These miRNAs might target species-specific genes, which could be validated in the future when the genome sequence and gene annotation of *C. gynandra* are available.

In this study, we explored the differences in the characteristics of miRNAs between *C. gynandra* and *C. hassleriana* using high-throughput sequencing technology. In addition to the identified known and novel miRNAs, the differentially expressed miRNAs and their functions were investigated in these two species. Because two different species were used, some of the observed differences in the miRNAs may be due to the differences in the genetic backgrounds rather than the differences between C_3_ and C_4_ photosynthesis. Future studies about the functions of these differentially expressed miRNAs will be helpful to clarify the question above and confirm the roles of these miRNAs in the process of photosynthesis. Our study not only provides a foundation for the further elucidation of miRNA function in *C. gynandra* and *C. hassleriana* but is also helpful for scientists to obtain some insights into the alteration in molecular pathways during the evolution of the C_3_ pathways to C_4_ pathways.

## Methods

### Plant material and RNA isolation

*C. gynandra* (also known as *Gynandropsis gynandra*) and *C. hassleriana* (also known as *Tarenaya hassleriana*) were grown in a standard potting mix in a 25 °C greenhouse under a 16:8 (light: dark) photoperiod. The fully expanded leaves (approximately 5 cm in length) were collected, immediately frozen in liquid nitrogen and stored at −80 °C. The total RNA was isolated from the leaves using TRIzol reagent (Life Technology, CA, USA) according to the manufacturer’s protocol. The total RNA concentration and purity were assayed with a NanoDrop ND-1000 spectrophotometer (NanoDrop Technologies, Wilmington, DE, USA). The RNA integrity was assessed on an Agilent 2100 Bioanalyzer Lab-on-Chip system (Agilent Technologies, Palo Alto, CA, USA). Small RNA fractions of 10–40 nucleotides were isolated from the total RNA pool with a Novex 15% TBE-urea gel (Invitrogen, USA).

### Library construction and high-throughput sequencing of small RNAs

The small RNA (sRNA) sequencing libraries were generated using the NEBNex Multiplex Small RNA Library Prep Set for Illumina (NEB, USA) following the manufacturer’s instructions. The libraries were quantified on the Agilent Bioanalyzer 2100 (Agilent Technologies, Waldbroon, Germany) system using DNA High Sensitivity Chips and were pooled so that each index-tagged sample was present in equimolar amounts. The clustering of the pooled index-coded samples was performed on a cBot Cluster Generation System using TruSeq SR Cluster Kit v3-cBot-HS (Illumia, USA) according to the manufacturer’s instructions. After the cluster generation, the libraries were sequenced on an Illumina HiSeq 2500 platform, and 50 bp single-end reads were generated. The dataset has been deposited in the National Center for Biotechnology Information (NCBI; accession number SRR5298259, SRR5298260).

### Data processing

The raw data (raw reads) were first processed through custom scripts. In this step, clean data (clean reads) were obtained by removing the reads that contained ploy-N, 3′adapter and insert tags, and low-quality reads from the raw data. The remaining unique reads were mapped to the *Arabidopsis thaliana* genome sequence by Bowtie2^42^. Then, the mapped sRNA reads were aligned with the Silva, GtRNAdb, Rfam and Repbase databases by Bowtie2 to remove the reads that originated from repeat sequences, rRNA, tRNA, snRNA, and snoRNA. The remaining clean unannotated reads could be used to detect the known and novel miRNAs.

The mapped clean unannotated sRNA reads were further used to identify the known miRNA with alignment against miRBase21.0 (http://www.mirbase.org/), mirdeep2^43^ and srna-tools-cli software. Matched sequences with no more than two mismatches were considered known miRNAs. The available software miREvo^44^ and mirdeep2 were integrated to predict novel miRNA through exploring the hairpin structure, the Dicer cleavage site and the minimum free energy of the small RNA unannotated reads.

### Expression and function of miRNAs

The MiRNA expression levels were estimated for each sample through the following steps: First, the sRNA sequences were mapped back onto the precursor sequence, the counts of the reads for each miRNA were obtained from the mapping results, and the TPM (transcripts per million) values of each miRNA were calculated^45^. The differential expression analysis of the miRNAs between the *C. gynandra* and *C. hassleriana* samples was performed using the DEseq^46^ R package. The P-value was adjusted using the q-value^47^. FDR ≤ 0.05 and |log2(foldchange)| ≥ 1 were set as the threshold for significant differential expression by default.

The prediction of the putative target genes of the miRNAs was performed by psRobot_tar in psRobot^48^ for plants based on a comparison with the annotations of *Arabidopsis* genome. A Gene Ontology (GO) enrichment analysis was used on the putative target genes of the miRNAs with the GOseq R packages based on the Wallenius non-central hyper-geometric distribution^49^. The KOBAS^50^ software was used to test the statistical enrichment of the putative target genes of the miRNAs in the KEGG pathways (http://www.genome.jp/kegg/)^51^.

### Differential gene expression profiling

The dataset of the publicly available RNA-seq data for the leaves of *C. gynandra* and *C. hassleriana* was downloaded from NCBI (accession number: SRP036637 and SRP036837)^19^. The differential expression analysis of genes between *C. gynandra* and *C. hassleriana* was performed using the DEGseq R package^52^. The P-value was adjusted using the q-value^47^. FDR ≤ 0.01 and |log2(foldchange)| ≥ 1 were set as the threshold for significant differential expression by default.

### Quantitative real-time PCR

To confirm the predicted results, qRT-PCR for miRNAs detection was performed to examine their expression. Fourteen miRNAs were randomly selected for the qRT-PCR assays in the samples of *C. gynandra* and *C. hassleriana* by Platinum SYBR Green-based qPCR (Invitrogen, USA) with the High-Specificity miRNA QuantiMir RT Kit (RA610A-1, System Biosciences) on the ViiA™ 7 Dx platform (ABI, USA). Amplified primers for all miRNAs were designed according to Varkonyi-Gasic *et al*.^53^. The miRNA-specific forward primers of the 14 selected miRNAs and the internal control U6 are listed in the supplementary table (Supplementary Table 5). The qRT-PCR procedure was as follows: 95 °C for 10 min, 40 cycles at 95 °C for 15 s and 60 °C for 30 s, and a final step at 95 °C for 15 s, 60 °C 1 min and 95 °C for 15 s. After the qRT-PCR amplification, the melting curve and amplification curve were examined to evaluate the specific amplification. The relative expression levels of the miRNAs were analyzed by the 2^−ΔΔct^ method, and U6 was used as the internal control. All qRT-PCR reactions were assayed in triplicates.

## Additional Information

**How to cite this article:** Gao, S. *et al*. Identification and characterization of miRNAs in two closely related C_4_ and C_3_ species of Cleome by high-throughput sequencing. *Sci. Rep.* **7**, 46552; doi: 10.1038/srep46552 (2017).

**Publisher's note:** Springer Nature remains neutral with regard to jurisdictional claims in published maps and institutional affiliations.

## Supplementary Material

Supplementary Information

## Figures and Tables

**Table 1:** Summary of the small RNA sequencing datasets.

RNA class	Cleome gynandra		Cleome hassleriana	
	Total	% of Total	Total	% of Total
Raw reads	6,698,595	100	7,177,444	100
3′ adapter and length filter	1,859,667	27.76	2,120,400	29.54
rRNA	2,917,851	43.56	3,139,295	43.74
snoRNA	562	0.01	1,419	0.02
tRNA	117,536	1.75	209,775	2.92
Repeats	41,425	0.62	19,347	0.27
Unannotated reads	1,761,554	26.30	1,687,208	23.51

**Table 2:** Known miRNAs identified in *Cleome gynandra* and *Cleome hassleriana*.

miRNA family	miRNA members	Reference miRNA of *Arabidopsis*	miRNA sequence	TPM	
				*Cleome gynandra*	*Cleome hassleriana*
MIR156	Cleome-miR156a-5p	ath-miR156a-5p	UGACAGAAGAGAGUGAGCAC	1791.13	361.33
	Cleome-miR156b-5p	ath-miR156b-5p	UGACAGAAGAGAGUGAGCAC	1787.50	359.85
	Cleome-miR156c-5p	ath-miR156c-5p	UGACAGAAGAGAGUGAGCAC	1791.13	361.33
	Cleome-miR156d-5p	ath-miR156d-5p	UGACAGAAGAGAGUGAGCAC	2103.58	352.48
	Cleome-miR156e	ath-miR156e	UGACAGAAGAGAGUGAGCAC	1776.60	352.48
	Cleome-miR156f-5p	ath-miR156f-5p	UGACAGAAGAGAGUGAGCAC	1776.60	352.48
	Cleome-miR156g	ath-miR156g	CGACAGAAGAGAGUGAGCAC	25.43	8.85
	Cleome-miR156h	ath-miR156h	UGACAGAAGAAAGAGAGCAC	0	7.374
	Cleome-miR156j	ath-miR156j	UGACAGAAGAGAGAGAGCAC	3.63	1.47
MIR157	Cleome-miR157a-3p	ath-miR157a-3p	GCUCUCUAGCCUUCUGUCAUC	0	1.4748
	Cleome-miR157a-5p	ath-miR157a-5p	UUGACAGAAGAUAGAGAGCAC	1733.00	5276.87
	Cleome-miR157b-3p	ath-miR157b-3p	GCUCUCUAGCCUUCUGUCAUC	0	1.4748
	Cleome-miR157b-5p	ath-miR157b-5p	UUGACAGAAGAUAGAGAGCAC	1733.00	5276.87
	Cleome-miR157c-5p	ath-miR157c-5p	UUGACAGAAGAUAGAGAGCAC	1827.46	5980.35
	Cleome-miR157d	ath-miR157d	UGACAGAAGAUAGAGAGCAC	316.08	511.76
MIR159	Cleome-miR159a	ath-miR159a	UUUGGAUUGAAGGGAGCUCUA	12091.05	20914.26
	Cleome-miR159b-3p	ath-miR159b-3p	UUUGGAUUGAAGGGAGCUCUU	2368.80	2325.77
	Cleome-miR159c	ath-miR159c	UUUGGAUUGAAGGGAGCUCCU	6081.85	556.00
MIR160	Cleome-miR160a-3p	ath-miR160a-3p	GCGUAUGAGGAGCCAUGCAUA	948.25	42.77
	Cleome-miR160a-5p	ath-miR160a-5p	UGCCUGGCUCCCUGUAUGCCA	1351.52	168.13
	Cleome-miR160b	ath-miR160b	UGCCUGGCUCCCUGUAUGCCA	1271.59	162.23
	Cleome-miR160c-5p	ath-miR160c-5p	UGCCUGGCUCCCUGUAUGCCA	1351.52	168.13
MIR161	Cleome-miR161.2	ath-miR161.2	UCAAUGCAUUGAAAGUGACUA	3.63	0
MIR162	Cleome-miR162a-3p	ath-miR162a-3p	UCGAUAAACCUCUGCAUCCAG	715.73	541.25
	Cleome-miR162a-5p	ath-miR162a-5p	UGGAGGCAGCGGUUCAUCGAUC	276.12	79.64
	Cleome-miR162b-3p	ath-miR162b-3p	UCGAUAAACCUCUGCAUCCAG	715.73	541.25
	Cleome-miR162b-5p	ath-miR162b-5p	UGGAGGCAGCGGUUCAUCGAUC	257.95	79.64
MIR164	Cleome-miR164a	ath-miR164a	UGGAGAAGCAGGGCACGUGCA	4781.19	9599.53
	Cleome-miR164b-5p	ath-miR164b-5p	UGGAGAAGCAGGGCACGUGCA	4879.29	9729.31
	Cleome-miR164c-5p	ath-miR164c-5p	UGGAGAAGCAGGGCACGUGCG	105.36	126.83
MIR165	Cleome-miR165a-3p	ath-miR165a-3p	UCGGACCAGGCUUCAUCCCCC	279.75	2572.07
	Cleome-miR165b	ath-miR165b	UCGGACCAGGCUUCAUCCCCC	279.75	2567.64
MIR166	Cleome-miR166a-3p	ath-miR166a-3p	UCGGACCAGGCUUCAUUCCCC	122665.26	119371.02
	Cleome-miR166b-3p	ath-miR166b-3p	UCGGACCAGGCUUCAUUCCCC	122483.61	119376.92
	Cleome-miR166c	ath-miR166c	UCGGACCAGGCUUCAUUCCCC	125281.11	136788.52
	Cleome-miR166d	ath-miR166d	UCGGACCAGGCUUCAUUCCCC	125281.11	136794.41
	Cleome-miR166e-3p	ath-miR166e-3p	UCGGACCAGGCUUCAUUCCCC	122549.00	119472.79
	Cleome-miR166e-5p	ath-miR166e-5p	GGAAUGUUGUCUGGCACGAGG	0	1.47
	Cleome-miR166f	ath-miR166f	UCGGACCAGGCUUCAUUCCCC	122545.37	119475.74
	Cleome-miR166g	ath-miR166g	UCGGACCAGGCUUCAUUCCCC	125346.51	136890.28
MIR167	Cleome-miR167a-5p	ath-miR167a-5p	UGAAGCUGCCAGCAUGAUCUA	22623.48	5222.30
	Cleome-miR167b	ath-miR167b	UGAAGCUGCCAGCAUGAUCUA	22568.98	5214.92
	Cleome-miR167c-5p	ath-miR167c-5p	UAAGCUGCCAGCAUGAUCUUG	14.53	4.42
	Cleome-miR167d	ath-miR167d	UGAAGCUGCCAGCAUGAUCUGG	4490.54	1278.66
MIR168	Cleome-miR168a-3p	ath-miR168a-3p	CCCGCCUUGCAUCAACUGAAU	406.91	622.37
	Cleome-miR168a-5p	ath-miR168a-5p	UCGCUUGGUGCAGGUCGGGAA	2786.61	6180.92
	Cleome-miR168b-5p	ath-miR168b-5p	UCGCUUGGUGCAGGUCGGGAA	2862.90	6310.71
MIR169	Cleome-miR169a-3p	ath-miR169a-3p	GGCAAGUUGUCCUUGGCUAC	0	8.85
	Cleome-miR169a-5p	ath-miR169a-5p	CAGCCAAGGAUGACUUGCCGA	3.63	8.85
	Cleome-miR169b-5p	ath-miR169b-5p	CAGCCAAGGAUGACUUGCCGG	174.39	8.85
	Cleome-miR169c	ath-miR169c	CAGCCAAGGAUGACUUGCCGG	130.79	0
	Cleome-miR169d	ath-miR169d	UGAGCCAAGGAUGACUUGCCG	0	1.47
	Cleome-miR169e	ath-miR169e	UGAGCCAAGGAUGACUUGCCG	0	1.47
	Cleome-miR169f-5p	ath-miR169f-5p	UGAGCCAAGGAUGACUUGCCG	47.23	1.47
	Cleome-miR169g-5p	ath-miR169g-5p	UGAGCCAAGGAUGACUUGCCG	43.60	1.47
MIR170	Cleome-miR170-5p	ath-miR170-5p	UAUUGGCCUGGUUCACUCAGA	72.66	58.99
MIR171	Cleome-miR171a-3p	ath-miR171a-3p	UGAUUGAGCCGCGCCAAUAUC	3.63	1.47
	Cleome-miR171a-5p	ath-miR171a-5p	UAUUGGCCUGGUUCACUCAGA	69.03	58.99
	Cleome-miR171b-3p	ath-miR171b-3p	UUGAGCCGUGCCAAUAUCACG	112.63	39.82
	Cleome-miR171c-3p	ath-miR171c-3p	UUGAGCCGUGCCAAUAUCACG	112.63	39.82
	Cleome-miR171c-5p	ath-miR171c-5p	AGAUAUUGGUGCGGUUCAAUC	36.33	28.02
MIR172	Cleome-miR172a	ath-miR172a	AGAAUCUUGAUGAUGCUGCAU	846.52	886.36
	Cleome-miR172b-3p	ath-miR172b-3p	AGAAUCUUGAUGAUGCUGCAU	846.52	886.36
	Cleome-miR172c	ath-miR172c	AGAAUCUUGAUGAUGCUGCAG	14.53	10.32
	Cleome-miR172d-3p	ath-miR172d-3p	AGAAUCUUGAUGAUGCUGCAG	14.53	8.85
	Cleome-miR172e-3p	ath-miR172e-3p	GGAAUCUUGAUGAUGCUGCAU	3.63	5.90
MIR2111	Cleome-miR2111a-3p	ath-miR2111a-3p	GUCCUCGGGAUGCGGAUUACC	14.53	1.47
	Cleome-miR2111a-5p	ath-miR2111a-5p	UAAUCUGCAUCCUGAGGUUUA	65.40	7.37
	Cleome-miR2111b-5p	ath-miR2111b-5p	UAAUCUGCAUCCUGAGGUUUA	61.76	4.42
MIR319	Cleome-miR319a	ath-miR319a	UUGGACUGAAGGGAGCUCCCU	185.29	1.47
	Cleome-miR319b	ath-miR319b	UUGGACUGAAGGGAGCUCCCU	207.09	1.47
	Cleome-miR319c	ath-miR319c	UUGGACUGAAGGGAGCUCCUU	18.17	47.19
MIR390	Cleome-miR390a-3p	ath-miR390a-3p	CGCUAUCCAUCCUGAGUUUCA	21.80	10.32
	Cleome-miR390a-5p	ath-miR390a-5p	AAGCUCAGGAGGGAUAGCGCC	1892.86	1259.49
	Cleome-miR390b-5p	ath-miR390b-5p	AAGCUCAGGAGGGAUAGCGCC	1892.86	1259.49
MIR393	Cleome-miR393a-5p	ath-miR393a-5p	UCCAAAGGGAUCGCAUUGAUCC	1169.87	373.13
	Cleome-miR393b-5p	ath-miR393b-5p	UCCAAAGGGAUCGCAUUGAUCC	1169.87	373.13
MIR394	Cleome-miR394a	ath-miR394a	UUGGCAUUCUGUCCACCUCC	461.41	168.13
	Cleome-miR394b-5p	ath-miR394b-5p	UUGGCAUUCUGUCCACCUCC	461.41	168.13
MIR395	Cleome-miR395a	ath-miR395a	CUGAAGUGUUUGGGGGAACUC	21.80	4.42
	Cleome-miR395b	ath-miR395b	CUGAAGUGUUUGGGGGGACUC	29.07	159.28
	Cleome-miR395c	ath-miR395c	CUGAAGUGUUUGGGGGGACUC	29.07	159.28
	Cleome-miR395d	ath-miR395d	CUGAAGUGUUUGGGGGAACUC	21.80	4.42
	Cleome-miR395e	ath-miR395e	CUGAAGUGUUUGGGGGAACUC	21.80	4.42
	Cleome-miR395f	ath-miR395f	CUGAAGUGUUUGGGGGGACUC	29.07	159.28
MIR396	Cleome-miR396a-3p	ath-miR396a-3p	GUUCAAUAAAGCUGUGGGAAG	1071.77	446.87
	Cleome-miR396a-5p	ath-miR396a-5p	UUCCACAGCUUUCUUGAACUG	5649.51	2558.79
	Cleome-miR396b-3p	ath-miR396b-3p	GCUCAAGAAAGCUGUGGGAAA	203.46	337.73
	Cleome-miR396b-5p	ath-miR396b-5p	UUCCACAGCUUUCUUGAACUU	1594.94	1402.54
MIR397	Cleome-miR397a	ath-miR397a	UCAUUGAGUGCAGCGUUGAUG	0	10.32
MIR398	Cleome-miR398a-3p	ath-miR398a-3p	UGUGUUCUCAGGUCACCCCUU	101.73	42.77
	Cleome-miR398b-3p	ath-miR398b-3p	UGUGUUCUCAGGUCACCCCUG	0	197.62
	Cleome-miR398c-3p	ath-miR398c-3p	UGUGUUCUCAGGUCACCCCUG	0	197.62
MIR399	Cleome-miR399b	ath-miR399b	UGCCAAAGGAGAGUUGCCCUG	18.17	30.97
	Cleome-miR399c-3p	ath-miR399c-3p	UGCCAAAGGAGAGUUGCCCUG	21.80	26.55
	Cleome-miR399d	ath-miR399d	UGCCAAAGGAGAUUUGCCCCG	7.27	17.70
	Cleome-miR399f	ath-miR399f	UGCCAAAGGAGAUUUGCCCGG	18.17	11.80
MIR403	Cleome-miR403-3p	ath-miR403-3p	UUAGAUUCACGCACAAACUCG	980.94	2153.22
MIR408	Cleome-miR408-3p	ath-miR408-3p	AUGCACUGCCUCUUCCCUGGC	18.17	1588.37
MIR8175	Cleome-miR8175	ath-miR8175	GAUCCCCGGCAACGGCGCCA	10.90	0
MIR824	Cleome-miR824-5p	ath-miR824-5p	UAGACCAUUUGUGAGAAGGGA	1860.16	920.28
MIR827	Cleome-miR827	ath-miR827	UUAGAUGACCAUCAACAAACU	559.50	244.82
MIR828	Cleome-miR828	ath-miR828	UCUUGCUUAAAUGAGUAUUCCA	0	1.47
MIR858	Cleome-miR858a	ath-miR858a	UUUCGUUGUCUGUUCGACCUU	185.29	535.36
	Cleome-miR858b	ath-miR858b	UUCGUUGUCUGUUCGACCUUG	610.37	536.83

**Table 3:** Novel miRNA candidates identified in *Cleome gynandra* and *Cleome hassleriana.*

miRNA name	miRNA sequence	TPM	
		*Cleome gynandra*	*Cleome hassleriana*
Cleome_novel_miR1	uuuccuucuucuuguugc	69.03	14.75
Cleome_novel_miR2	uucguccccacagacggcgcca	374.21	97.34
Cleome_novel_miR3	cgauccccggcaacggugcca	7.27	0
Cleome_novel_miR4	acaggugguggaacaaauaugagu	0	16.22

**Table 4:** Summary of the putative target genes of the miRNAs in *Cleome gynandra* and *Cleome hassleriana.*

Species	Number of all miRNAs	Number of miRNAs with Target genes	Number of Target genes
*Cleome gynandra*	97	68	228
*Cleome hassleriana*	105	72	223
Total	109	76	238
