# Supplementary material for: Identification and characterization of miRNAs in two closely related C4 and C3 species of Cleome by high-throughput sequencing
Source: Sci Rep. 2017 Apr 19;7:46552. doi: 10.1038/srep46552 (PMC5396198; doi:10.1038/srep46552)
Supplement: Supplementary Information [file srep46552-s1.pdf]

Identification and characterization of miRNAs in two closely related C_4 and C_3 species of Cleome by high-throughput sequencing

Shuangcheng Gao[*, 1]
Email: gsczml@163.com
Wei Zhao[*, 2]
Email: hustzwqq@163.com
Xiang Li[2]
Email: xiangli@scbg.ac.cn
Qingbo You[2]
Email: youqb2006@163.com
Xinjie Shen[2]
Email: ylssxj@163.com
Wei Guo[2],
Email: vivi1998@126.com
Shihua Wang[1]
Email: sshhww-11@163.com
Guoan Shi[1]
Email: gashi1963@163.com
Zheng Liu[#, 3]
Email: liuzhengxp@yeah.net
Yongqing Jiao[#, 2]
Email: jiaoyongqing@caas.cn

* These authors contributed equally to this work.
#Corresponding authors: jiaoyongqing@caas.cn; liuzhengxp@yeah.net
[1] College of Agriculture, Henan University of Science and Technology, Luoyang, Henan Province, 471003, P. R. China
[2] Key Laboratory of Biology and Genetic Improvement of Oil Crops, Ministry of Agriculture, Oil Crops Research Institute of the Chinese Academy of Agricultural Sciences, Wuhan, 430062, P. R. China
[3] College of Life Sciences, Hebei University, Baoding, Hebei Province, 071002, P. R. China

Supplementary Information

Additional supporting information may be found in the online version of this article:

Supplementary Table 1. Length abundance of the small RNA sequences in *Cleome gynandra* and *Cleome hassleriana*.

Supplementary Table 2. Differentially expressed miRNAs between *Cleome gynandra* and *Cleome hassleriana* (FDR≤0.05, Fold Change≥2).

Supplementary Table 3. Putative target genes of the miRNAs identified in *Cleome gynandra* and *Cleome hassleriana*.

Supplementary Table 4. The expression levels of the predicted target genes of the differentially expressed miRNAs between *Cleome gynandra* and *Cleome hassleriana*.

Supplementary Table 5. The validation primers for the miRNAs and U6 gene.

Supplementary Figure 1. Number of members of each miRNA family in *Cleome gynandra* and *Cleome hassleriana*.

Supplementary Table 1. Length abundance of the small RNA sequences in *Cleome gynandra* and *Cleome hassleriana*.

	Cleome gynandra				***Cleome hassleriana***			
**Length**	**Clean Reads**	**% of total Clean Reads**	**Mapped Reads**	**% of total Mapped Reads**	**Clean Reads**	**% of total Clean Reads**	**Mapped Reads**	**% of total Mapped Reads**
18	710,649	14.69%	5,217	4.84%	481,375	9.52%	5,826	2.50%
19	564,111	11.66%	6,830	6.34%	429,130	8.49%	7,265	3.12%
20	726,464	15.01%	11,748	10.91%	485,446	9.60%	10,583	4.55%
21	803,171	16.60%	60,066	55.78%	745,593	14.74%	141,052	60.58%
22	585,290	12.10%	7,181	6.67%	514,849	10.18%	7,821	3.36%
23	479,939	9.92%	3,948	3.67%	448,433	8.87%	5,029	2.16%
24	451,762	9.34%	3,051	2.83%	553,091	10.94%	5,057	2.17%
25	234,025	4.84%	2,201	2.04%	345,039	6.82%	5,179	2.22%
26	142,590	2.95%	2,184	2.03%	288,379	5.70%	9,925	4.26%
27	77,273	1.60%	944	0.88%	234,998	4.65%	5,860	2.52%
28	36,074	0.75%	543	0.50%	194,808	3.85%	4,535	1.95%
29	19,780	0.41%	3,698	3.43%	185,951	3.68%	22,842	9.81%
30	7,800	0.16%	79	0.07%	149,952	2.97%	1,860	0.80%
Total	4,838,928	100.00%	107,690	100.00%	5,057,044	100.00%	232,834	100.00%

Supplementary Table 2. Differentially expressed miRNAs between *Cleome gynandra* and *Cleome hassleriana* (FDR ≤ 0.05, Fold Change ≥ 2).

**miRNA family**	**miRNA name**	**TPM (*Cleome gynandra*)**	**TPM (*Cleome hassleriana*)**	**p-value**	**FDR**	**$\log_2$(Fold Change)**	**regulated**
MIR156	Cleome-miR156a-5p	1791.13	361.33	0	0	-2.31	down
	Cleome-miR156b-5p	1787.50	359.85	0	0	-2.31	down
	Cleome-miR156c-5p	1791.13	361.33	0	0	-2.31	down
	Cleome-miR156d-5p	2103.58	352.48	0	0	-2.58	down
	Cleome-miR156e	1776.60	352.48	0	0	-2.33	down
	Cleome-miR156f-5p	1776.60	352.48	0	0	-2.33	down
	Cleome-miR156g	25.43	8.85	1.0731E-02	1.4264E-02	-1.52	down
MIR157	Cleome-miR157a-5p	1733.00	5276.87	0	0	1.61	up
	Cleome-miR157b-5p	1733.00	5276.87	0	0	1.61	up
	Cleome-miR157c-5p	1827.46	5980.35	0	0	1.71	up
MIR159	Cleome-miR159c	6081.85	556.00	0	0	-3.45	down
MIR160	Cleome-miR160a-3p	948.25	42.77	0	0	-4.47	down
	Cleome-miR160a-5p	1351.52	168.13	0	0	-3.01	down
	Cleome-miR160b	1271.59	162.23	0	0	-2.97	down
	Cleome-miR160c-5p	1351.52	168.13	0	0	-3.01	down
MIR162	Cleome-miR162a-5p	276.12	79.64	0	0	-1.79	down
	Cleome-miR162b-5p	257.95	79.64	0	0	-1.70	down
MIR164	Cleome-miR164a	4781.19	9599.53	0	0	1.01	up
MIR165	Cleome-miR165a-3p	279.75	2572.07	0	0	3.20	up
	Cleome-miR165b	279.75	2567.64	0	0	3.20	up
MIR167	Cleome-miR167a-5p	22623.48	5222.30	0	0	-2.12	down
	Cleome-miR167b	22568.98	5214.92	0	0	-2.11	down
	Cleome-miR167c-5p	14.53	4.42	2.5271E-02	3.2030E-02	-1.72	down
	Cleome-miR167d	4490.54	1278.66	0	0	-1.81	down
MIR168	Cleome-miR168a-5p	2786.61	6180.92	0	0	1.15	up
	Cleome-miR168b-5p	2862.90	6310.71	0	0	1.14	up
MIR169	Cleome-miR169a-3p	0	8.85	3.7385E-02	4.3817E-02	26.40	up
	Cleome-miR169b-5p	174.39	8.85	0	0	-4.30	down
	Cleome-miR169c	130.79	0	0	0	-30.28	down
	Cleome-miR169f-5p	47.23	1.47	0	0	-5.00	down
	Cleome-miR169g-5p	43.60	1.47	1.0000E-06	1.8167E-06	-4.89	down
MIR171	Cleome-miR171b-3p	112.63	39.82	1.4000E-05	2.3659E-05	-1.50	down
	Cleome-miR171c-3p	112.63	39.82	1.4000E-05	2.3659E-05	-1.50	down
MIR2111	Cleome-miR2111a-3p	14.53	1.47	7.1360E-03	9.8459E-03	-3.30	down
	Cleome-miR2111a-5p	65.40	7.37	0	0	-3.15	down
	Cleome-miR2111b-5p	61.76	4.42	0	0	-3.80	down
MIR319	Cleome-miR319a	185.29	1.47	0	0	-6.97	down
	Cleome-miR319b	207.09	1.47	0	0	-7.13	down
	Cleome-miR319c	18.17	47.19	4.6500E-03	7.0396E-03	1.38	up

MIR390	Cleome-miR390a-3p	21.80	10.32	2.6436E-02	3.3121E-02	-1.08	down
MIR393	Cleome-miR393a-5p	1169.87	373.13	0	0	-1.65	down
	Cleome-miR393b-5p	1169.87	373.13	0	0	-1.65	down
MIR394	Cleome-miR394a	461.41	168.13	0	0	-1.46	down
	Cleome-miR394b-5p	461.41	168.13	0	0	-1.46	down
MIR395	Cleome-miR395a	21.80	4.42	5.0560E-03	7.3481E-03	-2.30	down
	Cleome-miR395b	29.07	159.28	0	0	2.45	up
	Cleome-miR395c	29.07	159.28	0	0	2.45	up
	Cleome-miR395d	21.80	4.42	5.0560E-03	7.3481E-03	-2.30	down
	Cleome-miR395e	21.80	4.42	5.0560E-03	7.3481E-03	-2.30	down
	Cleome-miR395f	29.07	159.28	0	0	2.45	up
MIR396	Cleome-miR396a-3p	1071.77	446.87	0	0	-1.26	down
	Cleome-miR396a-5p	5649.51	2558.79	0	0	-1.14	down
MIR397	Cleome-miR397a	0	10.32	2.6591E-02	3.2937E-02	26.62	up
MIR398	Cleome-miR398a-3p	101.73	42.77	1.7400E-04	2.7487E-04	-1.25	down
	Cleome-miR398b-3p	0	197.62	0	0	30.88	up
	Cleome-miR398c-3p	0	197.62	0	0	30.88	up
MIR399	Cleome-miR399d	7.27	17.70	3.6723E-02	4.3509E-02	1.28	up
MIR403	Cleome-miR403-3p	980.94	2153.22	0	0	1.13	up
MIR408	Cleome-miR408-3p	18.17	1588.37	0	0	6.45	up
MIR8175	Cleome-miR8175	10.90	0	6.9500E-03	9.7122E-03	-26.70	down
MIR824	Cleome-miR824-5p	1860.16	920.28	0	0	-1.02	down
MIR827	Cleome-miR827	559.50	244.82	0	0	-1.19	down
MIR858	Cleome-miR858a	185.29	535.36	0	0	1.53	up
NA	Cleome_novel_miR1	69.03	14.75	1.1000E-05	1.9032E-05	-2.23	down
NA	Cleome_novel_miR2	374.21	97.34	0	0	-1.94	down
NA	Cleome_novel_miR3	7.27	0	2.4070E-02	3.0866E-02	-26.11	down
NA	Cleome_novel_miR4	0	16.22	6.8060E-03	9.6345E-03	27.27	up

Supplementary Table 3. Putative target genes of the miRNAs identified in *Cleome gynandra* and *Cleome hassleriana*.

**miRNA family**	**miRNA name**	**Unique target gene number**	**Target Genes**
MIR156	Cleome-miR156a-5p	15	AT5G50570;AT1G71690;AT1G69170;AT5G43270;AT1G27360;AT5G50670;AT1G27370;AT2G42200;AT3G57920
	Cleome-miR156b-5p		AT1G69170;AT5G43270;AT1G27360;AT5G50570;AT1G71690;AT3G57920;AT5G50670;AT1G27370;AT2G42200
	Cleome-miR156c-5p		AT1G69170;AT5G43270;AT1G27360;AT5G50570;AT1G71690;AT3G57920;AT5G50670;AT1G27370;AT2G42200
	Cleome-miR156d-5p		AT5G50570;AT1G71690;AT1G69170;AT5G43270;AT1G27360;AT5G50670;AT1G27370;AT2G42200;AT3G57920
	Cleome-miR156e		AT1G71690;AT5G50570;AT5G43270;AT1G27360;AT1G69170;AT5G50670;AT2G42200;AT1G27370;AT3G57920
	Cleome-miR156f-5p		AT3G57920;AT2G42200;AT1G27370;AT5G50670;AT5G43270;AT1G27360;AT1G69170;AT1G71690;AT5G50570
	Cleome-miR156g		AT5G50670;AT2G42200;AT1G27370;AT3G57920;AT5G50570;AT5G43270;AT1G27360;AT1G69170
	Cleome-miR156h		AT5G50570;AT3G11960;AT1G69170;AT5G43270;AT1G22000;AT1G27360;AT1G27370;AT1G26890;AT2G42200;AT5G50670;AT5G38610;AT3G57920
	Cleome-miR156j		AT1G69170;AT1G22000;AT5G43270;AT1G27360;AT5G50570;AT1G71400;AT5G38610;AT3G57920;AT1G27370;AT1G26890;AT3G47170;AT2G42200;AT5G50670
MIR157	Cleome-miR157a-5p	11	AT1G69170;AT5G43270;AT1G27360;AT5G50570;AT5G08620;AT3G57920;AT1G27370;AT2G42200;AT5G50670
	Cleome-miR157b-5p		AT5G50670;AT2G42200;AT1G27370;AT3G57920;AT5G08620;AT5G50570;AT5G43270;AT1G27360;AT1G69170
	Cleome-miR157c-5p		AT2G42200;AT1G27370;AT5G50670;AT5G08620;AT3G57920;AT5G50570;AT1G27360;AT5G43270;AT1G69170
	Cleome-miR157d		AT3G57920;AT5G08620;AT5G50670;AT1G27370;AT2G42200;AT1G69170;AT5G43270;AT1G27360;AT1G30450;AT5G50570
MIR159	Cleome-miR159a	6	AT2G32460;AT4G37770;AT2G26950;AT2G34010
	Cleome-miR159b-3p		AT4G15530
	Cleome-miR159c		AT5G55020
MIR160	Cleome-miR160a-5p	3	AT1G77850;AT4G30080;AT2G28350
	Cleome-miR160b		AT4G30080;AT1G77850;AT2G28350
	Cleome-miR160c-5p		AT2G28350;AT4G30080;AT1G77850
MIR161	Cleome-miR161.2	13	AT1G62914;AT1G63330;AT1G63230;AT1G62590;AT1G62930;AT1G63630;AT5G16640;AT5G41170;AT5G65560;AT1G63130;AT1G62910;AT1G63150;AT1G63400
MIR164	Cleome-miR164a	6	AT1G56010;AT3G15170;AT3G12977;AT5G53950;AT5G07680;AT5G61430

	Cleome-miR164b-5p		AT5G53950;AT3G12977;AT5G61430;AT5G07680;AT3G15170;AT1G56010
	Cleome-miR164c-5p		AT5G07680;AT5G61430;AT3G12977;AT5G53950;AT1G56010;AT3G15170
MIR165	Cleome-miR165a-3p	4	AT5G60690;AT1G30490;AT4G32880;AT2G34710
	Cleome-miR165b		AT1G30490;AT5G60690;AT2G34710;AT4G32880
MIR166	Cleome-miR166a-3p	6	AT4G32880;AT2G34710;AT1G52150;AT5G60690;AT1G30490
	Cleome-miR166b-3p		AT4G32880;AT2G34710;AT5G60690;AT1G52150;AT1G30490
	Cleome-miR166c		AT5G60690;AT1G52150;AT1G30490;AT2G34710;AT4G32880
	Cleome-miR166d		AT4G32880;AT2G34710;AT5G60690;AT1G52150;AT1G30490
	Cleome-miR166e-3p		AT5G60690;AT1G52150;AT1G30490;AT2G34710;AT4G32880
	Cleome-miR166e-5p		AT5G65970
	Cleome-miR166f		AT1G30490;AT5G60690;AT1G52150;AT4G32880;AT2G34710
	Cleome-miR166g		AT2G34710;AT4G32880;AT1G30490;AT1G52150;AT5G60690
MIR167	Cleome-miR167d	1	AT3G61310
MIR169	Cleome-miR169a-3p	3	AT2G40840
	Cleome-miR169b-5p		AT5G42120
	Cleome-miR169c		AT5G42120
	Cleome-miR169d		AT1G70700
	Cleome-miR169e		AT1G70700
	Cleome-miR169f-5p		AT1G70700
	Cleome-miR169g-5p		AT1G70700
MIR171	Cleome-miR171a-3p	3	AT3G60630;AT4G00150;AT2G45160
	Cleome-miR171b-3p		AT3G60630;AT4G00150;AT2G45160
	Cleome-miR171c-3p		AT3G60630;AT2G45160;AT4G00150
MIR172	Cleome-miR172a	10	AT3G54990;AT2G28550;AT5G12900;AT5G60120;AT5G67180;AT5G65790;AT4G36920
	Cleome-miR172b-3p		AT5G60120;AT5G12900;AT3G54990;AT2G28550;AT4G36920;AT5G65790;AT5G67180
	Cleome-miR172c		AT5G60120;AT2G28550;AT3G54990;AT3G49690;AT4G36920;AT5G67180;AT2G39250
	Cleome-miR172d-3p		AT3G49690;AT4G36920;AT5G67180;AT2G39250;AT5G60120;AT2G28550;AT3G54990
	Cleome-miR172e-3p		AT3G54990;AT3G54350;AT5G60120;AT2G39250;AT5G67180;AT4G36920;AT5G65790
MIR2111	Cleome-miR2111a-5p	2	AT1G07010;AT3G27150
	Cleome-miR2111b-5p		AT1G07010;AT3G27150
MIR319	Cleome-miR319a	5	AT1G30210;AT4G18390
	Cleome-miR319b		AT1G30210;AT4G18390
	Cleome-miR319c		AT3G15030;AT2G31070;AT3G66658
MIR393	Cleome-miR393a-5p	4	AT3G23690;AT1G12820;AT3G62980;AT3G26810
	Cleome-miR393b-5p		AT1G12820;AT3G23690;AT3G26810;AT3G62980
MIR394	Cleome-miR394a	2	AT5G09670;AT1G27340
	Cleome-miR394b-5p		AT1G27340;AT5G09670

MIR395	Cleome-miR395a	5	AT5G13630;AT5G43780;AT2G28780;AT5G10180; AT3G22890
	Cleome-miR395b		AT2G28780;AT5G10180;AT5G43780
	Cleome-miR395c		AT2G28780;AT5G10180;AT5G43780
	Cleome-miR395d		AT2G28780;AT5G10180;AT5G43780;AT5G13630; AT3G22890
	Cleome-miR395e		AT5G10180;AT2G28780;AT5G43780;AT5G13630; AT3G22890
	Cleome-miR395f		AT5G43780;AT5G10180;AT2G28780
MIR396	Cleome-miR396a-5p	7	AT3G52910;AT2G36400
	Cleome-miR396b-3p		AT5G45070;AT5G07700
	Cleome-miR396b-5p		AT5G43060;AT5G01370;AT2G45480
MIR397	Cleome-miR397a	4	AT2G29130;AT2G38080;AT5G60020;AT5G18420
MIR408	Cleome-miR408-3p	1	AT2G02850
MIR824	Cleome-miR824-5p	1	AT3G57230
MIR828	Cleome-miR828	2	AT1G66370;AT5G52600
MIR858	Cleome-miR858a	7	AT5G49330;AT2G47460;AT5G35550;AT3G08500;AT4G12350;AT1G66230;AT1G06180
	Cleome-miR858b		AT1G66230;AT1G06180;AT3G08500;AT4G12350;AT5G49330;AT5G35550;AT2G47460
NA	Cleome_novel_miR1	129	AT4G38410;AT5G64230;AT2G30933;AT4G05200;AT2G41360;AT5G38860;AT5G42540;AT5G53170;AT1G56460;AT4G33580;AT2G44060;AT2G35920;AT3G20870;AT3G25230;AT3G29590;AT1G14610;AT2G07798;AT1G05085;AT3G02510;AT4G27590;AT3G13670;AT4G32800;AT1G29340;AT4G24880;AT1G63057;AT5G44650;AT5G02610;AT5G61330;AT4G30670;AT1G02670;AT3G10800;AT5G18140;AT1G36925;AT1G69500;AT4G01120;AT3G48390;AT1G19880;AT1G43640;AT4G11810;AT2G33793;AT1G35410;AT3G03300;AT4G21705;AT1G65810;AT4G29520;AT2G18290;AT5G13000;AT5G39570;AT2G33550;AT2G25670;AT1G19485;AT3G45690;AT3G22520;AT4G11730;AT2G22590;AT2G03230;AT1G58210;AT5G59220;AT4G16580;AT3G28720;AT1G63520;AT1G71080;AT5G11600;AT1G25410;AT2G20080;AT3G24740;AT3G07440;AT5G16280;AT1G01320;AT4G26450;AT3G61630;AT1G12860;AT3G22660;AT5G65490;AT5G64600;AT5G17080;AT3G52380;AT1G73020;AT1G53480;AT4G35440;AT5G65550;AT1G29600;AT5G51580;AT2G16940;AT5G17910;AT3G09500;AT4G03565;AT3G01770;AT1G35220;AT1G63680;AT5G62500;AT2G04680;AT3G01530;AT5G45510;AT4G00440;AT3G17420;AT3G59990;AT1G27170;AT1G51900;AT5G65830;AT2G25130;AT5G56360;AT4G25760;AT4G37490;AT2G01720;AT5G56200;AT5G63190;AT3G17680;AT5G11690;AT2G26190;AT1G53440;AT4G22320;AT1G56110;AT1G67670;AT5G57655;AT1G76180;AT4G15810;AT1G22530;AT2G42410;AT3G45130;AT5G25752;AT2G03130;AT1G63055;AT3G02930;AT1G03530;AT3G05935;AT1G27480;AT3G62630;AT1G29000
NA	Cleome_novel_miR3	1	AT5G20670

Supplementary Table 4. The expression levels of the predicted target genes of the differentially expressed miRNAs between *Cleome gynandra* and *Cleome hassleriana*.

miRNA name	*C. hassleriana* cds ID	AGI (*A. thaliana* orthologue ID)	RPKM_leaf_stage5 normalized (*C. hassleriana*) [a]	RPKM_leaf_stage5 normalized (*C. gynandra*) [b]	log_2 (Fold Change *C. hassleriana*/*C. gynandra*)	p-value	q-value	Accordance [c]
Cleome-miR156a-5p	T.hassleriana_13755	AT1G69170	20	0	5.311	3.67E-06	0.0000	Yes
Cleome-miR156a-5p	T.hassleriana_14530	AT2G42200	24	6	1.989	0.000731	0.0007	Yes
Cleome-miR156a-5p	T.hassleriana_06843	AT5G43270	76	28	1.430	1.93E-06	0.0000	Yes
Cleome-miR156a-5p	T.hassleriana_05028	AT5G50570	48	18	1.404	0.00019	0.0002	Yes
Cleome-miR156a-5p	T.hassleriana_15586	AT5G50570	43	6	2.831	2.69E-08	0.0000	Yes
Cleome-miR156b-5p	T.hassleriana_13755	AT1G69170	20	0	5.311	3.67E-06	0.0000	Yes
Cleome-miR156b-5p	T.hassleriana_14530	AT2G42200	24	6	1.989	0.000731	0.0007	Yes
Cleome-miR156b-5p	T.hassleriana_06843	AT5G43270	76	28	1.430	1.93E-06	0.0000	Yes
Cleome-miR156b-5p	T.hassleriana_05028	AT5G50570	48	18	1.404	0.00019	0.0002	Yes
Cleome-miR156b-5p	T.hassleriana_15586	AT5G50570	43	6	2.831	2.69E-08	0.0000	Yes
Cleome-miR156c-5p	T.hassleriana_13755	AT1G69170	20	0	5.311	3.67E-06	0.0000	Yes
Cleome-miR156c-5p	T.hassleriana_14530	AT2G42200	24	6	1.989	0.000731	0.0007	Yes
Cleome-miR156c-5p	T.hassleriana_06843	AT5G43270	76	28	1.430	1.93E-06	0.0000	Yes
Cleome-miR156c-5p	T.hassleriana_05028	AT5G50570	48	18	1.404	0.00019	0.0002	Yes
Cleome-miR156c-5p	T.hassleriana_15586	AT5G50570	43	6	2.831	2.69E-08	0.0000	Yes
Cleome-miR156d-5p	T.hassleriana_13755	AT1G69170	20	0	5.311	3.67E-06	0.0000	Yes
Cleome-miR156d-5p	T.hassleriana_14530	AT2G42200	24	6	1.989	0.000731	0.0007	Yes
Cleome-miR156d-5p	T.hassleriana_06843	AT5G43270	76	28	1.430	1.93E-06	0.0000	Yes
Cleome-miR156d-5p	T.hassleriana_05028	AT5G50570	48	18	1.404	0.00019	0.0002	Yes
Cleome-miR156d-5p	T.hassleriana_15586	AT5G50570	43	6	2.831	2.69E-08	0.0000	Yes
Cleome-miR156e	T.hassleriana_13755	AT1G69170	20	0	5.311	3.67E-06	0.0000	Yes
Cleome-miR156e	T.hassleriana_14530	AT2G42200	24	6	1.989	0.000731	0.0007	Yes
Cleome-miR156e	T.hassleriana_06843	AT5G43270	76	28	1.430	1.93E-06	0.0000	Yes
Cleome-miR156e	T.hassleriana_05028	AT5G50570	48	18	1.404	0.00019	0.0002	Yes
Cleome-miR156e	T.hassleriana_15586	AT5G50570	43	6	2.831	2.69E-08	0.0000	Yes
Cleome-miR156f-5p	T.hassleriana_13755	AT1G69170	20	0	5.311	3.67E-06	0.0000	Yes
Cleome-miR156f-5p	T.hassleriana_14530	AT2G42200	24	6	1.989	0.000731	0.0007	Yes
Cleome-miR156f-5p	T.hassleriana_06843	AT5G43270	76	28	1.430	1.93E-06	0.0000	Yes
Cleome-miR156f-5p	T.hassleriana_05028	AT5G50570	48	18	1.404	0.00019	0.0002	Yes
Cleome-miR156f-5p	T.hassleriana_15586	AT5G50570	43	6	2.831	2.69E-08	0.0000	Yes
Cleome-miR156g	T.hassleriana_13755	AT1G69170	20	0	5.311	3.67E-06	0.0000	Yes
Cleome-miR156g	T.hassleriana_14530	AT2G42200	24	6	1.989	0.000731	0.0007	Yes
Cleome-miR156g	T.hassleriana_06843	AT5G43270	76	28	1.430	1.93E-06	0.0000	Yes
Cleome-miR156g	T.hassleriana_05028	AT5G50570	48	18	1.404	0.00019	0.0002	Yes
Cleome-miR156g	T.hassleriana_15586	AT5G50570	43	6	2.831	2.69E-08	0.0000	Yes
Cleome-miR157a-5p	T.hassleriana_13755	AT1G69170	20	0	5.311	3.67E-06	0.0000	No
Cleome-miR157a-5p	T.hassleriana_14530	AT2G42200	24	6	1.989	0.000731	0.0007	No
Cleome-miR157a-5p	T.hassleriana_05028	AT5G50570	48	18	1.404	0.00019	0.0002	No
Cleome-miR157a-5p	T.hassleriana_15586	AT5G50570	43	6	2.831	2.69E-08	0.0000	No

Cleome-miR157b-5p	T.hassleriana_13755	AT1G69170	20	0	5.311	3.67E-06	0.0000	No
Cleome-miR157b-5p	T.hassleriana_14530	AT2G42200	24	6	1.989	0.000731	0.0007	No
Cleome-miR157b-5p	T.hassleriana_05028	AT5G50570	48	18	1.404	0.00019	0.0002	No
Cleome-miR157b-5p	T.hassleriana_15586	AT5G50570	43	6	2.831	2.69E-08	0.0000	No
Cleome-miR157c-5p	T.hassleriana_13755	AT1G69170	20	0	5.311	3.67E-06	0.0000	No
Cleome-miR157c-5p	T.hassleriana_14530	AT2G42200	24	6	1.989	0.000731	0.0007	No
Cleome-miR157c-5p	T.hassleriana_05028	AT5G50570	48	18	1.404	0.00019	0.0002	No
Cleome-miR157c-5p	T.hassleriana_15586	AT5G50570	43	6	2.831	2.69E-08	0.0000	No
Cleome-miR160a-5p	T.hassleriana_27921	AT2G28350	39	19	1.027	0.008641	0.0066	Yes
Cleome-miR160a-5p	T.hassleriana_14909	AT2G28350	17	0	5.077	2.15E-05	0.0000	Yes
Cleome-miR160b	T.hassleriana_27921	AT2G28350	39	19	1.027	0.008641	0.0066	Yes
Cleome-miR160b	T.hassleriana_14909	AT2G28350	17	0	5.077	2.15E-05	0.0000	Yes
Cleome-miR160c-5p	T.hassleriana_27921	AT2G28350	39	19	1.027	0.008641	0.0066	Yes
Cleome-miR160c-5p	T.hassleriana_14909	AT2G28350	17	0	5.077	2.15E-05	0.0000	Yes
Cleome-miR164a	T.hassleriana_16251	AT1G56010 (*NAC1*)	0	10	-4.333	0.001496	0.0014	Yes
Cleome-miR165a-3p	T.hassleriana_09295	AT2G34710 (*PHB*)	54	7	2.937	2.19E-10	0.0000	No
Cleome-miR165a-3p	T.hassleriana_18265	AT5G60690 (*REV*)	97	36	1.419	8.9E-08	0.0000	No
Cleome-miR165a-3p	T.hassleriana_19132	AT5G60690 (*REV*)	54	17	1.657	7.82E-06	0.0000	No
Cleome-miR165b	T.hassleriana_09295	AT2G34710 (*PHB*)	54	7	2.937	2.19E-10	0.0000	No
Cleome-miR165b	T.hassleriana_18265	AT5G60690 (*REV*)	97	36	1.419	8.9E-08	0.0000	No
Cleome-miR165b	T.hassleriana_19132	AT5G60690 (*REV*)	54	17	1.657	7.82E-06	0.0000	No
Cleome-miR171b-3p	T.hassleriana_17363	AT2G45160	91	45	1.005	8.09E-05	0.0001	Yes
Cleome-miR171b-3p	T.hassleriana_07704	AT3G60630	22	71	-1.701	1.6E-07	0.0000	No
Cleome-miR171c-3p	T.hassleriana_17363	AT2G45160	91	45	1.005	8.09E-05	0.0001	Yes
Cleome-miR171c-3p	T.hassleriana_07704	AT3G60630	22	71	-1.701	1.6E-07	0.0000	No
Cleome-miR319c	T.hassleriana_27785	AT2G31070	18	165	-3.207	3.94E-31	0.0000	Yes
Cleome-miR319c	T.hassleriana_15699	AT2G31070	104	257	-1.316	1.64E-16	0.0000	Yes
Cleome-miR319c	T.hassleriana_09477	AT3G15030	110	54	1.016	1.24E-05	0.0000	No
Cleome-miR394a	T.hassleriana_10278	AT1G27340	101	8	3.648	1.55E-21	0.0000	Yes
Cleome-miR394b-5p	T.hassleriana_10278	AT1G27340	101	8	3.648	1.55E-21	0.0000	Yes
Cleome-miR395a	T.hassleriana_00457	AT5G10180 (*AST68*)	60	18	1.726	1.23E-06	0.0000	Yes
Cleome-miR395a	T.hassleriana_28828	AT5G13630 (*GUN5*)	1790	4148	-1.223	4.1E-215	0.0000	No
Cleome-miR395a	T.hassleriana_00325	AT3G22890 (*APS1*)	93	28	1.721	1.68E-09	0.0000	Yes
Cleome-miR395b	T.hassleriana_00457	AT5G10180 (*AST68*)	60	18	1.726	1.23E-06	0.0000	No
Cleome-miR395c	T.hassleriana_00457	AT5G10180 (*AST68*)	60	18	1.726	1.23E-06	0.0000	No
Cleome-miR395d	T.hassleriana_00457	AT5G10180 (*AST68*)	60	18	1.726	1.23E-06	0.0000	Yes
Cleome-miR395d	T.hassleriana_28828	AT5G13630 (*GUN5*)	1790	4148	-1.223	4.1E-215	0.0000	No
Cleome-miR395d	T.hassleriana_00325	AT3G22890 (*APS1*)	93	28	1.721	1.68E-09	0.0000	Yes
Cleome-miR395e	T.hassleriana_00457	AT5G10180 (*AST68*)	60	18	1.726	1.23E-06	0.0000	Yes
Cleome-miR395e	T.hassleriana_28828	AT5G13630 (*GUN5*)	1790	4148	-1.223	4.1E-215	0.0000	No
Cleome-miR395e	T.hassleriana_00325	AT3G22890 (*APS1*)	93	28	1.721	1.68E-09	0.0000	Yes
Cleome-miR395f	T.hassleriana_00457	AT5G10180 (*AST68*)	60	18	1.726	1.23E-06	0.0000	No
Cleome-miR396a-5p	T.hassleriana_05324	AT2G36400	11	0	4.449	0.00084	0.0008	Yes
Cleome-miR397a	T.hassleriana_27873	AT2G29130	31	6	2.359	1.96E-05	0.0000	No

Cleome-miR858a	T.hassleriana_00009	AT1G66230	59	17	1.785	8.55E-07	0.0000	No
Cleome_novel_miR1	T.hassleriana_04134	AT1G19485	42	16	1.382	0.000565	0.0006	Yes
Cleome_novel_miR1	T.hassleriana_13548	AT1G29000	1	11	-3.470	0.001952	0.0017	No
Cleome_novel_miR1	T.hassleriana_01835	AT1G56110	140	61	1.188	2.18E-08	0.0000	Yes
Cleome_novel_miR1	T.hassleriana_14728	AT1G56110	155	61	1.335	1.11E-10	0.0000	Yes
Cleome_novel_miR1	T.hassleriana_09506	AT1G56110	183	52	1.805	2.41E-18	0.0000	Yes
Cleome_novel_miR1	T.hassleriana_24305	AT1G63680	122	60	1.013	4.36E-06	0.0000	Yes
Cleome_novel_miR1	T.hassleriana_01391	AT1G63680	12	0	4.574	0.00045	0.0005	Yes
Cleome_novel_miR1	T.hassleriana_06440	AT1G65810	10	29	-1.547	0.001758	0.0016	No
Cleome_novel_miR1	T.hassleriana_17016	AT1G73020	17	62	-1.877	1.58E-07	0.0000	No
Cleome_novel_miR1	T.hassleriana_24939	AT2G01720	96	41	1.217	2.31E-06	0.0000	Yes
Cleome_novel_miR1	T.hassleriana_27274	AT2G18290	79	7	3.486	1.18E-16	0.0000	Yes
Cleome_novel_miR1	T.hassleriana_20865	AT2G18290	87	7	3.625	1.08E-18	0.0000	Yes
Cleome_novel_miR1	T.hassleriana_22379	AT2G26190	82	34	1.259	7.27E-06	0.0000	Yes
Cleome_novel_miR1	T.hassleriana_06837	AT2G33550	1	23	-4.534	1.13E-06	0.0000	No
Cleome_novel_miR1	T.hassleriana_23253	AT2G33550	18	60	-1.748	9.1E-07	0.0000	No
Cleome_novel_miR1	T.hassleriana_23264	AT2G33793	1	38	-5.259	1.56E-10	0.0000	No
Cleome_novel_miR1	T.hassleriana_14713	AT2G44060	498	183	1.434	2.75E-34	0.0000	Yes
Cleome_novel_miR1	T.hassleriana_16195	AT3G13670	74	32	1.199	4.15E-05	0.0001	Yes
Cleome_novel_miR1	T.hassleriana_08545	AT3G17420	21	2	3.382	2.45E-05	0.0000	Yes
Cleome_novel_miR1	T.hassleriana_05224	AT3G24740	183	38	2.257	5.37E-24	0.0000	Yes
Cleome_novel_miR1	T.hassleriana_07768	AT3G59990	17	5	1.755	0.009009	0.0068	Yes
Cleome_novel_miR1	T.hassleriana_14720	AT3G59990	96	25	1.931	3.53E-11	0.0000	Yes
Cleome_novel_miR1	T.hassleriana_17587	AT3G62630	82	16	2.347	4.42E-12	0.0000	Yes
Cleome_novel_miR1	T.hassleriana_07904	AT4G01120	59	7	3.065	1.27E-11	0.0000	Yes
Cleome_novel_miR1	T.hassleriana_14468	AT4G03565	171	0	8.407	5.1E-36	0.0000	Yes
Cleome_novel_miR1	T.hassleriana_05412	AT4G15810	70	23	1.595	7.33E-07	0.0000	Yes
Cleome_novel_miR1	T.hassleriana_11126	AT4G21705	106	26	2.017	7.92E-13	0.0000	Yes
Cleome_novel_miR1	T.hassleriana_22837	AT4G22320	36	17	1.072	0.008975	0.0068	Yes
Cleome_novel_miR1	T.hassleriana_25414	AT4G22320	55	13	2.070	1.54E-07	0.0000	Yes
Cleome_novel_miR1	T.hassleriana_11457	AT4G26450	37	10	1.877	5.47E-05	0.0001	Yes
Cleome_novel_miR1	T.hassleriana_06982	AT4G33580	209	50	2.053	2.61E-24	0.0000	Yes
Cleome_novel_miR1	T.hassleriana_03397	AT4G35440	85	35	1.270	4.32E-06	0.0000	Yes
Cleome_novel_miR1	T.hassleriana_14231	AT5G17910	22	8	1.449	0.009696	0.0072	Yes
Cleome_novel_miR1	T.hassleriana_22699	AT5G45510	13	0	4.690	0.000242	0.0003	Yes
Cleome_novel_miR1	T.hassleriana_15451	AT5G56360	45	13	1.781	1.77E-05	0.0000	Yes
Cleome_novel_miR1	T.hassleriana_19056	AT5G59220	19	2	3.237	8.16E-05	0.0001	Yes
Cleome_novel_miR1	T.hassleriana_16598	AT5G59220	39	1	5.275	1.04E-10	0.0000	Yes
Cleome_novel_miR1	T.hassleriana_26959	AT5G62500	28	9	1.627	0.001489	0.0014	Yes
Cleome_novel_miR1	T.hassleriana_08680	AT5G62500	54	8	2.744	8.67E-10	0.0000	Yes
Cleome_novel_miR1	T.hassleriana_00481	AT5G65490	22	5	2.127	0.000735	0.0007	Yes
Cleome_novel_miR1	T.hassleriana_00194	AT5G65490	48	7	2.767	6.38E-09	0.0000	Yes
Cleome_novel_miR1	T.hassleriana_26803	AT5G65550	4	21	-2.403	0.000363	0.0004	No

71 [a] The expression level of genes of mature leaves at stage 5 in *C. hassleriana* from Külahoglu et al. (2014).

[b] The expression level of genes of mature leaves at stage 5 in *C. gynandra* from Külahoglu et al. (2014).

[c] Represented whether the changes in the miRNA and mRNA expression were in opposite directions.

Supplementary Table 5. The validation primers for the miRNAs and U6 gene.

miRNA ID	miRNA-specific forward primer sequence
Cleome-miR157a-3p	GCGGCGGGCTCTCTAGCCTTCT
Cleome-miR157a-5p	GCGGCGGTTGACAGAAGATAGA
Cleome-miR162b-5p	GCGGCGGTGGAGGCAGCGGTTCA
Cleome-miR164a	GCGGCGGTGGAGAAGCAGGGCA
Cleome-miR165b	GCGGCGGTCGGACCAGGCTTCA
Cleome-miR168a-5p	GCGGCGGTCGCTTGGTGCAGGT
Cleome-miR169d	GCGGCGGTGAGCCAAGGATGAC
Cleome-miR170-5p	GCGGCGGTATTGGCCTGGTTCA
Cleome-miR171b-3p	GCGGCGGTTGAGCCGTGCCAAT
Cleome-miR172b-3p	GCGGCGGAGAATCTTGATGATG
Cleome-miR390a-5p	GCGGCGGAAGCTCAGGAGGGAT
Cleome-miR393a-5p	GCGGCGGTCCAAAGGGATCGCAT
Cleome-miR395f	GCGGCGGCTGAAGTGTTTGGGG
Cleome-miR399c-3p	GCGGCGGTGCCAAAGGAGAGTT
U6	TGGCCCCTGCGCAAGGATGA

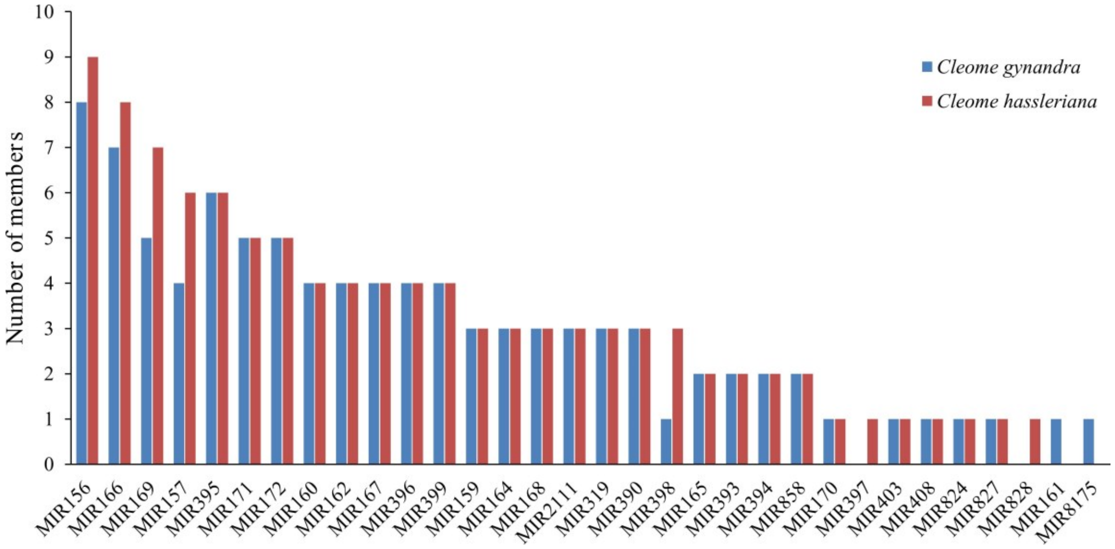

Supplementary Figure 1. Number of members of each miRNA family in *Cleome gynandra* and *Cleome hassleriana*.
